# Identifying dietary patterns related to metabolic syndrome using the 7th Korea National Health and Nutrition Examination Survey

**DOI:** 10.1017/S0007114525103905

**Published:** 2025-07-28

**Authors:** Jee Yeon Hong, Yu-Mi Kim, Min-Ho Shin, Sang-Baek Koh, Hyeon Chang Kim, Mi Kyung Kim

**Affiliations:** 1 Department of Preventive Medicine, College of Medicine, Hanyang University, Seoul, Republic of Korea; 2 Institute for Health and Society, Hanyang University, Seoul, Republic of Korea; 3 Department of Preventive Medicine, Chonnam National University Medical School, Gwangju, Republic of Korea; 4 Department of Preventive Medicine and Institute of Occupational Medicine, Yonsei University Wonju College of Medicine, Wonju, Republic of Korea; 5 Department of Preventive Medicine, Yonsei University College of Medicine, Seoul, Republic of Korea

**Keywords:** Metabolic syndrome, Dietary patterns, Surveys and questionnaires, Regression analysis, Chronic disease

## Abstract

Metabolic syndrome (MetS) is associated with an increased risk of CVD, type 2 diabetes and death from all causes. Dietary factors correlate with MetS, making diet a potential target for intervention. We used data from the 2012–2016 Korea National Health and Nutrition Examination Survey (KNHANES, *n* 12 122) to identify a dietary pattern (DP) using thirty-nine predefined food groups as predictors. MetS components were used as the response variable with the food groups in reduced rank regression followed by stepwise linear regression analyses. We then verified the Korean status of the DP externally in the Cardiovascular Disease Association Study (CAVAS) (*n* 8277) and the Health EXAminees (HEXA) study (*n* 48 610). The DP score, which included twenty food groups, showed significant positive associations with all MetS components and a higher prevalence ratio in KNHANES participants (*P* < 0·0001). Although the score was NS in CAVAS (*P* = 0·0913), it showed a strong positive association with MetS prevalence in HEXA (*P* < 0·0001). We identified and tested a DP associated with MetS in Korean populations. This DP may be a useful tool for assessing MetS risk. Although the score was linked to higher MetS risk, particularly in the predominantly urban population of the HEXA study, further validation in more diverse populations is needed.

## Graphical abstract

**Figure:**
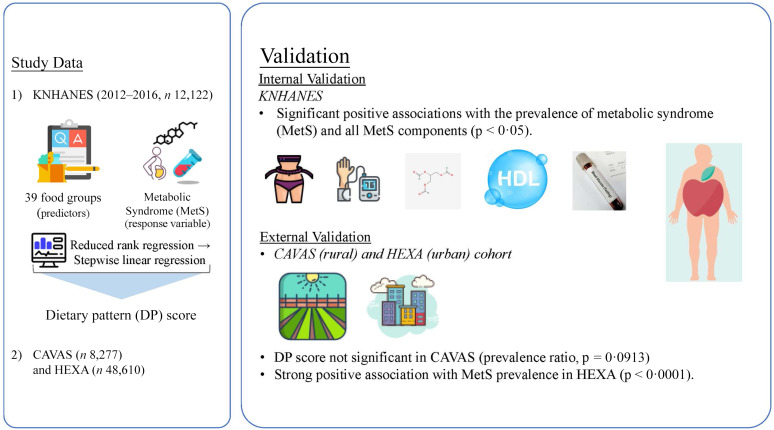

Metabolic syndrome (MetS) is a cardiometabolic risk factor associated with increased risk of cardiovascular disease^(1)^, type 2 diabetes^(2)^ and death from all causes^(1)^. MetS is defined by elevated measurements of three or more of five indicators: waist circumference (WC), blood pressure and levels of fasting blood glucose (FBG), HDL-cholesterol and TAG^3)^. Epidemiological evidence indicates that MetS is a highly prevalent disease worldwide, occurring in 20–25 % of the adult population^(4)^. Thus, MetS presents an urgent challenge to human health that profoundly affects the national health care system. Clinical and scientific studies have suggested that changing lifestyle, such as diet, can be an effective strategy in reducing the risk of developing MetS^(5–8)^. Thus, a better understanding of dietary factors associated with MetS risk may benefit human health.

A systematic review of dietary patterns (DPs) and MetS showed that healthy (prudent) DPs, which were developed mostly by an empirically dietary data-driven approach such as factor analysis, were inversely associated with MetS^(9)^. However, a new approach using reduced rank regression (RRR) was applied to develop a DP based on a dietary data-driven approach combined with diet–disease association^(10)^. A DP identified through RRR was found to be significantly linked to the prevalence of MetS in the overall population^(11)^. RRR generates a linear blend of food groups that effectively accounts for fluctuations in intermediate response variables associated with diseases^(12)^. Consequently, RRR is valuable in examining particular hypotheses concerning the potential pathways, specifically intermediate risk factors, through which diet could impact disease outcomes^(13)^. However, a DP related to MetS has not been identified using RRR in the Korea National Health and Nutrition Examination Survey (KNHANES), or verified externally using a DP score in the Cardiovascular Disease Association Study (CAVAS) and the Health EXAminees (HEXA) study. Therefore, our goal was to use RRR to formulate a DP with optimal correspondence to the variability in the components of MetS in a dataset that represents the Korean people and to check whether the DP reflects various Korean populations.

## Materials and methods

### Study design and population

To identify the DP, we used the 2012–2016 KNHANES^(14)^, which is a cross-sectional, but nationally representative survey. The KNHANES was designed to investigate health and nutritional status and consists of health interviews, health examinations and a nutritional survey, including an FFQ. From the 2012–2016 KNHANES, a total of 17 553 participants aged 20–65 years were included.

The DP was applied to two cohort studies from the Korean Genome and Epidemiology Study^(15)^: the CAVAS, a rural population-based prospective study to identify the risk factors for cardiovascular disease, and the HEXA, a prospective urban population-based study that was established to investigate the etiological factors of complex diseases. Participants in these two cohorts were examined repeatedly during the follow-up. The CAVAS enrolled participants aged ≥ 40 years between 2005 and 2011 who were asked to revisit within 2–4 years between 2007 and 2013. The proportion who revisited the survey through 2013 was 78·19 %. In the present study, 15 283 participants who revisited in the first re-examination were included. The HEXA cohort, which was established between 2003 and 2013, enrolled 167 169 urban residents (aged 40–79 years) and re-examined only 65 642 of them between 2012 and 2016. After excluding centres that were involved in the pilot study, had different data collection processes or participated for less than 2 years, 64 485 individuals who had completed the follow-up survey were included in this study.

We initially selected participants who did not have a history of heart disease, stroke or cancer, and we excluded those who were diagnosed with these conditions between visits (KNHANES *n* 14 964; CAVAS *n* 14 420; HEXA *n* 57 641). Additionally, we excluded participants who met any of the following criteria: (1) prior physician diagnosis of hypertension (taking antihypertensive agents) or type 2 diabetes (hypoglycaemic treatment: oral agents or insulin), and, additionally, participants aged ≥ 80 years at the second visit in the CAVAS and HEXA (KNHANES *n* 2137; CAVAS *n* 5201; HEXA *n* 5776); participants (2) who initially or upon revisiting the survey were missing > ten food items or had implausible dietary intakes (< 0·5 % or > 99·5 % of total energy intake) (KNHANES *n* 2; CAVAS *n* 644; HEXA *n* 553); (3) with missing values for MetS components (KNHANES *n* 465; CAVAS *n* 87; HEXA *n* 22) and (4) who had missing covariate values, such as for BMI, education level, regular exercise, smoking status or alcohol consumption (KNHANES *n* 343; CAVAS *n* 211; HEXA *n* 2700). After these exclusions, the development set had 12 122 participants from KNHANES (4559 men and 7563 women), 8277 participants from CAVAS (3158 men and 5119 women) and 48 590 participants from HEXA (15 602 men and 32 988 women) as shown in online Supplementary Figure 1.

### Assessment of dietary data

Dietary data in the KNHANES and CAVAS cohorts were assessed with their respective FFQ, which were administered by trained interviewers. In the KNHANES, the semi-quantitative FFQ contained 112 food items, including three alcoholic beverages. The KNHANES FFQ was conducted by well-trained interviewers^(16)^. The frequency of intake values was available in nine categories (never or less than once per month, once per month, 2–3 times per month, once per week, 2–4 times per week, 5–6 times per week, once per day, 2 times per day and 3 times per day). Some dairy products had additional questions about milk type (whole milk, low-fat or similar milk). Fruit items had questions regarding seasonal consumption (3, 6, 9 or 12 months of the year) as well as their frequency. Coffee items required an additional response if respondents drank coffee three or more times per day. For amounts, three serving sizes (0·5×, 1·0× and 1·5× standard portion size) were possible. As an exception, staple Korean foods such as total rice, other rice and *gimbap*, included a 4th serving category (0·5×, 1·0×, 1·5× or 2× standard portion size). Daily food consumption was calculated using weighted frequencies per day and average servings per occasion for each food. Among the 112 food items, three alcoholic beverages were excluded. Additionally, the KNHANES questionnaire was conducted to answer ‘butter/margarine’ and ‘jam’ with loaf bread, and ‘coffee sugar’ and ‘coffee cream’ with coffee. Therefore, we separated these two food items into six food items, which created four new additional food items, leading to a total of 113 food items.

In the CAVAS and the HEXA study of the Korean Genome and Epidemiology Study, a validated FFQ that was composed of 106 food items was used to assess dietary intake by trained interviewers (twice in CAVAS and once in HEXA)^(17)^. The FFQ used in the Korean Genome and Epidemiology Study was different from that of the KNHANES, but the structures including frequency (nine categories), average portion size (but 0·5×, 1·0× or 1·5× standard portion size for staple foods) and the exceptions for seasonal foods were similar. Although the present study focused on individuals who completed the first re-examination, we calculated the average food consumption (servings/day) based on dietary data from both the initial and the first follow-up visit to reduce measurement errors in the dietary assessment^(18)^.

Before the analysis for the development of DMetSI, we first grouped the food items into food groups considering the similarity between nutrient content and culinary use. For fruits and vegetables, colour, edible parts and botanical families were additionally considered^(19–22)^. Vegetables were classified into pickled and non-pickled vegetables first, and then pickled vegetables were classified into two different types of *kimchi* according to their edible parts and color^(23)^. Single food items reflecting a particular eating habit or food items that were already combined to account for eating habits were individually regarded as a single food group (for example, grain powder, cornflakes, tomatoes, mushrooms and pizzas/hamburgers). A detailed explanation of the food grouping of the FFQ used in the Korean Genome and Epidemiology Study (both CAVAS and HEXA) is described in a previous article^(24)^. However, food items of the KNHANES were also grouped in the same way as the CAVAS and HEXA. Three food groups were created in individual populations. For the KNHANES: (1) other rice: *bibimbab*/fried rice, *gimbab*, curry rice; (2) garlic and (3) fruit juice. For the CAVAS and HEXA: (1) dark yellow vegetables: carrot/carrot juice; pumpkin/sweet pumpkin/pumpkin juice; (2) Shellfish: clam/whelk and oysters and (3) other beverages. A total of thirty-nine food groups, but not the same food groups, were used in each population. The detailed list of food items and food groups is provided in online Supplementary Table 1.

### Assessment of non-dietary covariates

In all three populations, well-trained interviewers and examiners collected data, strictly following their own standardised protocols for the questionnaire surveys and examinations. Non-dietary covariates such as age, sex, years of education, smoking status, alcohol consumption and physical activity were collected. The following definitions were used in the final analyses: education levels (high school graduates or less); regular exercise (≥ 5 times per week or ≥ 30 min per session (KNHANES) or ≥ 3 times per week and ≥ 30 min per session (CAVAS and HEXA)) and smoking status (current smoker or non-smoker). The total alcohol consumption (ml/d) was represented by the total volume of alcohol consumed per day, which was calculated from the responses on the average frequencies and average amount of alcoholic beverages commonly consumed in Korea during the preceding year (six alcoholic beverages – *soju*, *makgeolli* (*takju*, unrefined rice wine), beer, *cheongju* (refined rice wine), wine and spirits in the CAVAS and HEXA. Three alcoholic beverages (*soju*, beer and *makgeolli* (*takju*, unrefined rice wine)) were included in the KNHANES).

### Definition of five individual metabolic abnormalities and metabolic syndrome

Anthropometric data and BP measurements in the KNHANES and CAVAS were obtained in a similar way. Height was measured to the nearest 0·1 cm using a standard height scale, and weight was measured with a metric weight scale to the nearest 0·01 kg, with the patient wearing light clothing without shoes. The BMI was calculated as weight (kg)/height (m^2^). WC was measured at the midpoint between the bottom of the rib margin and the iliac crest. Systolic blood pressure (SBP) and diastolic blood pressure (DBP) measurements were taken twice with a 5-min resting period from the right arm by auscultation using a standard mercury sphygmomanometer or an automatic sphygmomanometer. If there was a 5 mmHg or more difference between the first two consecutive readings, blood pressure were measured once more. The arithmetic mean was used in the analysis. Biochemical data, such as FBG, HDL-cholesterol and TAG, were determined in serum collected after fasting for at least 8 h. The analysis was conducted using a Hitachi Automatic Analyzer 7600–210 (Hitachi/JAPAN) for the KNHANES or an ADVIA 1650 Automated Analyzer (Siemens, New York, NY, USA) for the CAVAS and HEXA.

We defined MetS based on cardiovascular risk factors, such as abdominal obesity, high BP, high FBG level, low HDL-cholesterol and hypertriglyceridaemia, with measurements elevated to ≥ 3. The definition of MetS was based on criteria from the National Cholesterol Education Program Adult Treatment Panel III report^(3)^. We also modified other criteria of the Korean Diabetes Association criteria for WC and the International Diabetes Federation criteria for FBG^(25)^: (1) WC ≥ 90 cm in men and ≥ 85 cm in women^(26)^, (2) SBP ≥ 130 mmHg or DBP ≥ 85 mmHg or taking antihypertensive agents, (3) FBG ≥ 100 mg/dl or taking antidiabetic medication and (4) TG ≥ 150 mg/dl, (5) HDL-cholesterol < 40 mg/dl in men and < 50 mg/dl in women.

### Statistical analysis

All statistical analyses were performed using SAS, version 9.4 (SAS Institute, Inc.). Study characteristics are presented as mean and standard deviation for the continuous variables and as frequency and percentage for the categorical variables (online Supplementary Table 2). Statistical significance was set at *P* < 0·05.

To identify the DP based on food groups that were relevant to the MetS components, we performed a two-step approach. The first step was conducted using the RRR model to identify the linear function of the thirty-nine predefined food groups (servings/day). This explains variations in the components of MetS (WC, SBP, DBP, HDL-cholesterol, TAG and FBG in continuous form)^(10,12)^ in the KNHANES. In this step, we extracted the first factor (RRR DP score). The second step was stepwise linear regression analyses using the RRR DP score as the dependent variable and the thirty-nine food groups (servings/day) as independent variables to identify the most significant food groups contributing to the RRR DP score (significance level of *P* = 0·05 for entry and retention). The DP scores were calculated by weighting the selected food groups (servings/day) from the stepwise linear regression with their regression coefficients and then summing them in the KNHANES, CAVAS and HEXA studies.

Spearman’s correlation coefficients were used to evaluate the associations between DP score, selected foods and components of MetS in the KNHANES. To understand how the MetS components reflected the DP, the comparisons of average general characteristics and MetS components according to quintiles of the DP score were conducted using a generalised linear model and Tukey’s post hoc comparison test in the KNHANES. The prevalence ratio (PR) and 95 % CI of MetS according to the quintiles of the DP score (using the lowest quintile (Q1) as the reference group) was obtained by a generalised linear model in the KNHANES, CAVAS and HEXA. The models were as follows: model 1, the unadjusted model; model 2, the age and sex (in total)-adjusted model and model 3, the covariates-adjusted (age, sex, BMI, education level, regular exercise, current smoking, alcohoconsumption and total energy intake) model. The median values of each quintile group were treated as a continuous variable for the analysis of the linear trend.

This study was conducted in accordance with the guidelines in the Declaration of Helsinki and was approved by the Institutional Review Board of Hanyang University (IRB No: HYUIRB-202104-017-3). All participants provided written informed consent before participating in the study.

## Results

Compared with participants in the CAVAS and HEXA cohorts, those in the KNHANES cohort were younger (38·92 (sd 0·16) years *v*. 58·86 (sd 0·10) and 51·37 (sd 0·02) years, respectively; *P* < 0·001) and had higher proportions of men (49·56 % *v*. 38·15 % and 32·11 %; *P* < 0·001), high school graduates (72·59 % *v*. 30·82 % and 69·66 %; *P* < 0·001), regular exercisers (41·75 % *v*. 23·80 % and 34·43 %; *P* < 0·001) and current smokers (24·12 % *v*. 13·72 % and 12·91 %; *P* < 0·001). Participants in the KNHANES also showed higher alcohol consumption (18·39 (sd 0·42) g/d) than those in the CAVAS (11·13 (sd 0·34) g/d) and HEXA (8·73 (sd 0·09) g/d; *P* < 0·001). Energy intake was also highest in the KNHANES (2164·96 (sd 10·68) kcal/d), followed by HEXA (1700·09 (sd 1·55)) and CAVAS (1586·76 (sd 5·23)); *P* < 0·001 for the total. Metabolic syndrome (MetS) prevalence was highest in the CAVAS (24·15 %), followed by KNHANES (14·88 %) and HEXA (13·90 %; *P* < 0·001) (online Supplementary Table 2).

A total of twenty food groups were selected to derive the DP score using RRR followed by stepwise linear regression in the KNHANES (Table 1). Among these, eleven food groups had positive weights (garlic, carbonated beverages, nuts, noodles, total rice, tea, legumes, green leaf *kimchi*, coffee additives, white root *kimchi* and fish), and nine had negative weights (milk, fruit, potatoes starch, mushrooms, spreads, dessert, cornflake, pizza/hamburger and rice cakes).

**Table 1.** Selected food groups and the weights of the dietary pattern (DP) in the Korea National Health and Nutrition Examination Survey (KNHANES)*

Food group	Weight (*β*)
Garlic	0·4178
Carbonated beverages	0·3881
Nuts	0·3678
Noodles	0·3094
Total rice	0·2842
Tea	0·1454
Legumes	0·0982
*Kimchi*, Green Leaf	0·0649
Coffee additives	0·0638
*Kimchi*, White Root	0·0562
Fish	0·038
Milk	–0·0888
Fruit	–0·1146
Potatoes starch	–0·1480
Mushrooms	–0·1775
Spreads	–0·3503
Dessert	–0·3632
Cornflake	–0·3873
Pizza/Hamburger	–0·7531
Rice Cakes	–0·7543

* DP = ∑[(weight of each food group) × (individual intake of food groups as serving size)].

The correlation analysis revealed that the DP score was significantly associated with all components of MetS in KNHANES. Specifically, it showed positive correlations with WC (*β* = 0·674), SBP (*β* = 0·659), DBP (*β* = 0·667), TAG (*β* = 0·601) and FBG (*β* = 0·451) and a negative correlation with HDL-cholesterol (*r* = –0·487) (online Supplementary Table 3).

Across quintiles of the DP score in total population from the KNHANES, the prevalence of MetS increased progressively from 10·63 % in the lowest quintile to 21·17 % in the highest (*P*-trend < 0·0001, online Supplementary Table 4–1). Higher DP scores were associated with being older (mean age: 32·95 (sd 0·24) in Q1 *v*. 44·39 (sd 0·24) in Q5, *P*_trend_ < 0·0001) and having a higher proportion of men (15·64 % in Q1 *v*. 81·39 % in Q5, *P*_trend_ < 0·0001). BMI also increased significantly (22·79 (sd 0·08) to 23·68 (sd 0·07) kg/m^2^, *P*_trend_ < 0·0001). Educational attainment (≥12 years) declined with higher DP score quintile (75·13 % in Q1 *v*. 71·20 % in Q5, *P*_trend_ = 0·0058), as did regular physical activity (45·78 % in Q1 *v*. 32·99 % in Q5, *P*_trend_ < 0·0001). In contrast, current smoking (14·09 % to 34·02 %) and alcohol intake (12·05 (sd 0·74) g/d to 25·96 (sd 0·71) g/d) increased significantly across quintiles (*P*_trend_ < 0·0001 for both). Total energy intake also increased (2035·35 (sd 18·20) kcal to 2251·39 (sd 17·63) kcal, *P*_trend_ < 0·0001). Covariate trends in the CAVAS (online Supplementary Table 4–2) and HEXA (online Supplementary Table 4–3) studies mirrored those in KNHANES (*P*-trend < 0·0001), supporting the reproducibility of the DP. The tendency of the covariates was the same in the DP score quintiles of all three populations except for age. Similar patterns were observed in BMI, education level ≥ 12 years, regular exercise status, proportion of current smokers and alcohol consumption. The prevalence of MetS was high in all the highest quintiles of the DP score.

Table 2 shows the adjusted means of MetS components and the prevalence of MetS across quintiles of the DP score in the KNHANES population, based on model 3, which includes the most comprehensive adjustments. Across DP quintiles, all components of MetS exhibited significant linear trends (*P*_trend_ < 0·05), indicating that a higher DP score was consistently associated with a worsened cardiometabolic profile (*P*_trend_ < 0·05). The prevalence of MetS increased from 13·68 % in Quintile 1 to 18·65 % in Quintile 5, showing a significant linear trend (*P*_trend_ < 0·0001). Also, the PR of MetS according to the DP score increased significantly (PR (95 % CI), *P*_trend_: 1·66 (1·33, 2·07)).

**Table 2.** The comparison of means of the metabolic syndrome components and prevalence of the metabolic syndrome according to quintile of the dietary pattern (DP) in the Korea National Health and Nutrition Examination Survey (KNHANES)* (Mean values and standard deviations or Mean values with their standard errors; prevalence ratios and 95 % confidence intervals)

	Quintile 1		Quintile 2		Quintile 3		Quintile 4		Quintile 5		*P*-diff†	*P*-trend‡
	Mean	sd/se	Mean	sd/se	Mean	sd/se	Mean	sd/se	Mean	sd/se		
*n*	2424		2424		2424		2424		2424			
Median	0·03		0·44		0·71		0·98		1·42			
Min, Max	–3·50, 0·28		0·28, 0·57		0·57, 0·83		0·83, 1·15		1·15, 4·22			
WC												
Model 1§	75·34	9·85^a^	77·99	9·85^b^	79·97	9·85^c^	81·89	9·35^d^	83·69	9·35^e^	< 0·0001	< 0·0001
Model 2||	77·67	0·20^a^	78·82	0·19^b^	79·66	0·19^c^	80·09	0·19^c^	80·22	0·19^c^	< 0·0001	< 0·0001
Model 3¶	79·06	0·09	79·25	0·09	79·39	0·09	79·34	0·09	79·40	0·09	0·0709	0·0148
SBP												
Model 1§	108·13	13·29^a^	111·63	13·29^b^	113·52	13·29^c^	114·98	13·29^d^	117·63	12·80^e^	< 0·0001	< 0·0001
Model 2||	111·42	0·28^a^	112·99	0·26^b^	113·55	0·26^bc^	113·41	0·26^bc^	114·28	0·27^c^	< 0·0001	< 0·0001
Model 3¶	112·15	0·26^a^	113·29	0·25^b^	113·53	0·25^b^	113·08	0·25^ab^	113·62	0·26^b^	0·0006	0·0014
DBP												
Model 1§	71·26	9·85^a^	73·72	9·85^b^	75·19	9·85^c^	76·38	9·35^d^	78·57	9·35^e^	< 0·0001	< 0·0001
Model 2||	73·68	0·20^a^	74·69	0·19^b^	75·13	0·19^b^	75·07	0·19^b^	75·88	0·20^c^	< 0·0001	< 0·0001
Model 3¶	74·19	0·19^a^	74·91	0·19^b^	75·14	0·18^b^	74·82	0·18^ab^	75·41	0·19^b^	0·0003	0·0002
TG												
Model 1§	95·71	111·27^a^	113·57	111·76^b^	125·44	111·27^c^	138·78	108·32^d^	166·21	103·88^e^	< 0·0001	< 0·0001
Model 2||	113·39	2·35^a^	120·37	2·25^ab^	124·23	2·22^b^	127·54	2·21^b^	143·74	2·27^c^	< 0·0001	< 0·0001
Model 3¶	120·74	2·26^a^	124·74	2·16^a^	125·24	2·13^ab^	124·26	2·12^a^	133·45	2·23^b^	0·0023	0·0005
HDL-C												
Model 1§	56·03	12·31^a^	54·41	12·31^b^	52·00	12·31^c^	50·23	11·82^d^	48·57	11·32^e^	< 0·0001	< 0·0001
Model 2||	54·00	0·25^a^	53·73	0·24^a^	52·38	0·24^b^	52·01	0·24^b^	51·95	0·24^b^	< 0·0001	< 0·0001
Model 3¶	53·42	0·24^ab^	53·58	0·23^a^	52·58	0·23^bc^	52·38	0·22^c^	52·02	0·24^c^	< 0·0001	< 0·0001
FBG												
Model 1§	90·42	15·26^a^	92·24	15·26^b^	93·80	15·26^c^	95·40	14·77^d^	97·16	14·28^e^	< 0·0001	< 0·0001
Model 2||	92·98	0·32^a^	93·42	0·31^a^	94·13	0·31^ab^	94·82	0·31^bc^	95·59	0·31^c^	< 0·0001	< 0·0001
Model 3¶	93·63	0·32^a^	93·70	0·30^a^	94·11	0·30^ab^	94·49	0·30^ab^	94·99	0·31^b^	0·0250	0·0013
MetS prevalence												
Model 1§	5·74^a^		9·94^b^		14·47^c^		18·10^d^		24·79^e^		< 0·0001	< 0·0001
Model 2||	10·63^a^		12·12^a^		14·92^b^		16·61^b^		21·17^c^		< 0·0001	< 0·0001
Model 3¶	13·68^a^		13·36^a^		14·71^a^		15·07^a^		18·65^b^		< 0·0001	< 0·0001
			PR	95 % CI	PR	95 % CI	PR	95 % CI	PR	95 % CI		
MetS prevalence ratio												
Model 1§	1·00 (ref.)		1·73	1·38, 2·17	2·52	2·04, 3·12	3·15	2·56, 3·88	4·32	3·54, 5·27		< 0·0001
Model 2||	1·00 (ref.)		1·44	1·15, 1·8	1·86	1·5, 2·32	2·05	1·65, 2·54	2·44	1·97, 3·04		< 0·0001
Model 3¶	1·00 (ref.)		1·23	1, 1·52	1·42	1·16, 1·73	1·44	1·17, 1·76	1·66	1·33, 2·07		< 0·0001

* Values are expressed as means (sd) for unadjusted model (model 1) and means (se) for adjusted models (models 2 and 3).

† *P* values for differences were determined using a general linear model (Tukey’s multiple comparison). Values within a row with different superscript letters (a, b, c, d, e) are significantly different from each other at p < 0.05.

‡ *P* values for linear trends were constructed by treating the median value of each group as a continuous variable.

§ Unadjusted model.

|| Adjusted for age and sex.

¶ Adjusted for age, sex, BMI, education level, regular exercise, current smoking, alcohol consumption and total energy intake.

In CAVAS, the association between the DP score and MetS prevalence was not statistically significant in the fully adjusted model 3 (PR: 1·13 (95 % CI: 0·99, 1·28), *P*_trend_ = 0·0913 in total population; 1·09 (0·90, 1·31), *P* = 0·3252 in men; 1·04 (0·88, 1·21), *P* = 0·4479 in women) (Table 3). In contrast, the association was robust and consistent in the HEXA study. The adjusted PR of MetS across DP quintiles were 1·40 (1·33, 1·47) in total, 1·20 (1·13, 1·29) in men and 1·31 (1·23, 1·40) in women, with *P*_trend_ < 0·0001 for all.

**Table 3.** The applying the dietary pattern (DP) in the Cardiovascular Disease Association Study (CAVAS) and the Health EXAminees (HEXA) study by the comparison of the prevalence ratio of the metabolic syndrome* (Prevalence ratios and 95 % confidence intervals)

	CAVAS						HEXA					
	Quintile 1	Quintile 3		Quintile 5		*P*-trend*	Quintile 1	Quintile 3		Quintile 5		*P*-trend*
		PR	95 % CI	PR	95 % CI			PR	95 % CI	PR	95 % CI	
Total												
* n*	1655	1655		1655			9722	9722		9722		
Median	0·56	1·05		1·51			0·32	0·92		1·50		
Min, Max	–1·56, 0·75	0·97, 1·13		1·34, 4·36			–4·54, 0·54	0·82, 1·03		1·28, 6·73		
Model 1†	1·00 (ref.)	1·15	1·01, 1·31	1·32	1·17, 1·49	< 0·0001	1·00 (ref.)	1·46	1·39, 1·54	1·97	1·88, 2·07	< 0·0001
Model 2‡	1·00 (ref.)	1·13	0·99, 1·28	1·34	1·18, 1·52	< 0·0001	1·00 (ref.)	1·32	1·25, 1·38	1·63	1·55, 1·71	< 0·0001
Model 3§	1·00 (ref.)	1·04	0·92, 1·18	1·13	0·99, 1·28	0·0913	1·00 (ref.)	1·24	1·18, 1·31	1·40	1·33, 1·47	< 0·0001
Men												
* n*	631	632		631			3120	3120		3120		
Median	0·82	1·22		1·69			0·63	1·14		1·72		
Min, Max	–1·56, 0·97	1·14, 1·30		1·51, 4·36			–2·85, 0·83	1·05, 1·24		1·50, 6·46		
Model 1†	1·00 (ref.)	1·01	0·83, 1·22	1·21	1·00, 1·45	0·0086	1·00 (ref.)	1·13	1·06, 1·21	1·31	1·23, 1·40	< 0·0001
Model 2‡	1·00 (ref.)	0·99	0·82, 1·21	1·15	0·95, 1·39	0·0439	1·00 (ref.)	1·13	1·06, 1·21	1·31	1·23, 1·40	< 0·0001
Model 3§	1·00 (ref.)	0·98	0·81, 1·18	1·09	0·90, 1·31	0·3252	1·00 (ref.)	1·11	1·04, 1·19	1·20	1·13, 1·29	< 0·0001
Women												
* n*	1023	1024		1024			6598	6598		6598		
Median	0·46	0·95		1·34			0·24	0·82		1·34		
Min, Max	–1·19, 0·65	0·86, 1·03		1·21, 2·45			–4·54, 0·45	0·72, 0·92		1·15, 6·73		
Model 1†	1·00 (ref.)	1·28	1·09, 1·51	1·31	1·11, 1·54	< 0·0001	1·00 (ref.)	1·36	1·27, 1·45	1·67	1·57, 1·78	< 0·0001
Model 2‡	1·00 (ref.)	1·17	0·99, 1·38	1·24	1·05, 1·46	0·0008	1·00 (ref.)	1·31	1·22, 1·40	1·64	1·54, 1·75	< 0·0001
Model 3§	1·00 (ref.)	1·05	0·90, 1·23	1·04	0·88, 1·21	0·4479	1·00 (ref.)	1·20	1·12, 1·28	1·31	1·23, 1·40	< 0·0001

* *P* values for linear trends were constructed by treating the median value of each group as a continuous variable.

† Crude model.

‡ Adjusted for age and sex (in total).

§ Adjusted for sex (in total), age, BMI, education level, regular exercise, current smoking, alcohol consumption and total energy intake.

## Discussion

To the best of our knowledge, this is the first article that has identified a DP related to MetS components. This pattern, derived from twenty food groups, was obtained using the RRR method and data from the KNHANES. Participants with higher DP scores exhibited elevated levels of WC, SBP, DBP, TAG and FBG and lower HDL-cholesterol. The DP score reflected the prevalence of the MetS in the KNHANES and HEXA cohorts, but not in the CAVAS cohort.

Some nutrients and foods are known to be linked to individual components of MetS^(27)^. However, to prevent or treat MetS, dietary advice focusing on overall DPs can be a better approach than single-nutrient interventions^(28)^. The twenty food groups selected for the DP were as follows: eleven food groups (total rice, noodles, legumes, nuts, garlic, green leaf *kimchi*, white root *kimchi,* fish, carbonated beverages, tea and coffee additives) were considered the risk-associated group and nine food groups (rice cakes, cornflakes, potatoes starch, dessert, spreads, mushrooms, fruit, milk and pizza/hamburger) were considered the protective group. The overall tendency between food groups and health outcome was similar to previous findings^(9,24,29)^: (1) animal-based foods (e.g. fish); salty foods (e.g. legumes, green leaf *kimchi* and white root *kimchi*); refined grains (total rice and noodles) and sugary foods (e.g. carbonated beverages and coffee additives) tended to be risk-associated food groups and (2) plant-based foods (e.g. potatoes starch, mushrooms and fruit); dairy products (e.g. milk) and whole grains (e.g. cornflakes) tended to be protective food groups.

However, some food groups were inconsistent with previous studies. First, among the positive labelled foods, tea (weight) showed a positive association with MetS components. To date, the relationship between tea and MetS components remains uncertain due to conflicting evidence. A recent prospective cohort study suggested that increased tea consumption may burden liver metabolism and lead to adverse effects in certain circumstances due to the large amount of herbal extracts of tea^(30)^. However, a review article has suggested that tea consumption may reduce the risk of MetS^(31)^. Furthermore, several prospective studies have found that other components in green tea, such as Fe and Cu, have been identified as harmful oxidant-inducing metals that increase the risk of type 2 diabetes^(32,33)^. This suggests that the dietary role of copper in tea can have a positive association with MetS. However, it has also been suggested that tea consumption can reduce the risk of MetS^(31)^. The second food is nuts. Although *Aspergillus* in nuts may produce potentially harmful compounds called mycotoxins^(34)^, nuts are a well-known beneficial food in individuals with MetS^(35)^. The third food is garlic, which is also known to have a protective role in MetS^(36)^, although it has been reported that the allicin in garlic can cause liver toxicity if taken in large quantities^(37)^. In Korea, garlic is a common ingredient normally eaten in the form of *kimchi* or with a variety of side dishes. However, we could not assert whether any participants had an excessive intake of garlic that could lead to liver toxicity. Garlic used in the present analysis was the consumption of garlic itself, for example, when consumed together with grilled red meat and not as a seasoning. Thus, we could not rule out the possibility that the DP associated with garlic consumption, beyond its use as a seasoning, may affect liver health. Given that the potential disruption to normal insulin signalling pathways that regulate lipid and glucose metabolism^(38)^ could result in altered levels of TAG, HDL-cholesterol and FBG and that they also demonstrated beneficial effects, we could not explain the unexpected direction of these three foods in the present study.

Some of the foods were also unexpectedly negatively correlated. First, we found an inverse relationship for rice cakes, spreads and desserts with MetS risk in our analysis, despite their high content of refined grains, sugar and fat. These results may be partially explained by the fact that rice cakes in Korea normally contain red beans or nuts, which might have a protective effect^(39)^. Therefore, there could be some beneficial substances even in desserts and spreads such as strawberry jam^(24)^. Next, pizzas/hamburgers (a single food item in the present study), which are recognised as unhealthy fast foods in Korea and deemed to have risks associated with inflammatory reactions^(11)^, showed an unexpected inverse relationship with MetS risk. However, although the anti-inflammatory role of pizza may be due to tomato paste, which contains the anti-inflammatory nutrient lycopene^(40)^, which is 2·5–4 times more bioavailable than the lycopene from fresh tomatoes^(41)^, participants in the present study consumed very little and thus it was also grouped into the non-traditional DP by hierarchical cluster analysis with Spearman’s correlation coefficient of servings/day (data not shown). The DP score may be a useful index reflecting the overall effect of foods related to MetS components rather than the effect of a single food. However, the RRR analysis of Korean DPs linked to MetS components did not clearly distinguish between healthy and unhealthy food groups. This could be due to interrelationships among different foods. This suggests that further research that includes food-wide analyses is needed to derive Korean DPs that consider interrelationships between foods.

In addition to possible biological mechanisms discussed above, several unexpected findings in this study may be explained by limitations in the methodology and data interpretation. First, the RRR method identifies DPs that explain the maximum variance in response variables, but it does not necessarily separate foods into clear categories of ‘healthy’ and ‘unhealthy.’ The derived DP score reflects statistical associations rather than direct causal relationships. Second, residual confounding may exist due to unmeasured covariates, which may affect the observed associations. Third, some food items such as desserts, spreads or pizza/hamburgers are mixed dishes with complex ingredients (e.g. nuts, red beans and tomato paste) that may exert mixed metabolic effects. Their inverse associations with MetS may reflect low overall consumption levels, reverse causality or cultural preparation patterns in Korea. Lastly, measurement errors in food frequency data and misclassification of food groupings based on 24-h recall or FFQ data are inherent limitations in most nutritional epidemiology studies. These limitations warrant careful interpretation and further validation in longitudinal cohorts using more precise dietary assessment tools.

The DP showed significant associations with the multivariable-adjusted model in the KNHANES (Table 2) and HEXA cohort but not in the CAVAS population (Table 3). Although KNHANES surveyed households selected across the country to represent the entire nation, the differences in population characteristics of the two cohorts may explain why the DPs identified here do not apply in the CAVAS cohort and apply in the HEXA cohort. First, the average age of the CAVAS cohort was almost 60 years old and the prevalence of MetS exceeded 24 %, whereas the HEXA cohort was relatively young, around 40–50 years old and the prevalence of MetS was less than 15 %. Second, a previous study showed a difference in nutrient intake and risk of MetS in CAVAS and HEXA^(42)^. CAVAS residents had higher carbohydrate and Na consumption and a higher risk of MetS compared with HEXA residents. The highest quartile subjects of carbohydrate and Na intake were more likely to develop MetS. This difference in DPs was also found in the KNHANES. There is a higher intake of fat, vitamins, fruits and fish and higher dietary quality^(43,44)^, but a lower intake of rice and *kimchi* in urban areas than in rural areas^(45)^. This finding indicates that even DPs from a national sample may still vary by age group or region, suggesting the need to consider these differences when developing preventive strategies.

Several limitations should be noted when interpreting the results of our study. First, the present study findings may not apply to those with other dietary cultures, although the identification of DP can be applied to other populations. Second, the KNHANES is a cross-sectional survey, and thus single FFQ data and MetS components measured at the same time point were used, therefore measurement error can exist. Third, our DP was identified using MetS components as response variables, but this approach may lead to the omission of DPs related to MetS but not related to other important intermediate response variables of interest^(46)^. Fourth, there were no standard values for comparing the DP score, and therefore recommended standards should be established^(47)^ with cut-offs associated with MetS occurrence. Lastly, foods that take a different direction than expected can lead to challenges in making recommendations and can also be a limitation. Nevertheless, to the best of our knowledge, the DP score in the present study represents a unique DP using the dietary consumption of foods and MetS components obtained from a community-based study.

In conclusion, we discovered a DP composed of food groups that explain the variability of MetS components, and it was internally and externally verified. The DP may apply to residents in urban areas. However, before these indices can be applied in public health and clinical settings to predict the risk of MetS and other MetS-related diseases such as diabetes and cardiovascular disease, further clarification of food items in Korean DPs and validation in different populations is needed.

## Supporting information

Hong et al. supplementary material Hong et al. supplementary material

## Supplementary material

For supplementary material/s referred to in this article, please visit https://doi.org/10.1017/S0007114525103905
