# Supplementary material for: Identifying dietary patterns related to metabolic syndrome using the 7th Korea National Health and Nutrition Examination Survey
Source: Br J Nutr. 2025 Jul 14;134(2):147–55. doi: 10.1017/S0007114525103905 (PMC12433745; doi:10.1017/S0007114525103905)
Supplement: Hong et al. supplementary material [file S0007114525103905sup001.docx]

Supplementary Materials: Identifying dietary patterns related to metabolic syndrome using the 7th Korea national health and nutrition examination survey

Jee Yeon Hong^1,2^, Yu-Mi Kim^1,2^, Min-Ho Shin^3^, Sang-Baek Koh^4^, Hyeon Chang Kim^5^, Mi Kyung Kim^1,2^*

*^1^ Department of Preventive Medicine, College of Medicine, Hanyang University, Seoul, Republic of Korea*

*^2^ Institute for Health and Society, Hanyang University, Seoul, Republic of Korea*

*^3^ Department of Preventive Medicine, Chonnam National University Medical School, Gwangju, Republic of Korea*

*^4^ Department of Preventive Medicine and Institute of Occupational Medicine, Yonsei University Wonju College of Medicine, Wonju, Republic of Korea*

*^5^ Department of Preventive Medicine, Yonsei University College of Medicine, Seoul, Republic of Korea*

* Corresponding author:

Mi Kyung Kim

Department of Preventive Medicine, Medical School Building A-Room 517-2, Department of Preventive Medicine, Hanyang University, College of Medicine, 222 Wangsimni-ro, Sungdong-Gu, Seoul, 04763, Republic of Korea

Phone: +82-2-2220-0667; Fax: +82-2-2293-0660; Email: [kmkkim@hanyang.ac.kr](mailto:kmkkim@hanyang.ac.kr)

**Supplemental Figure 1.** Flow charts of identifying and applying sets of dietary pattern


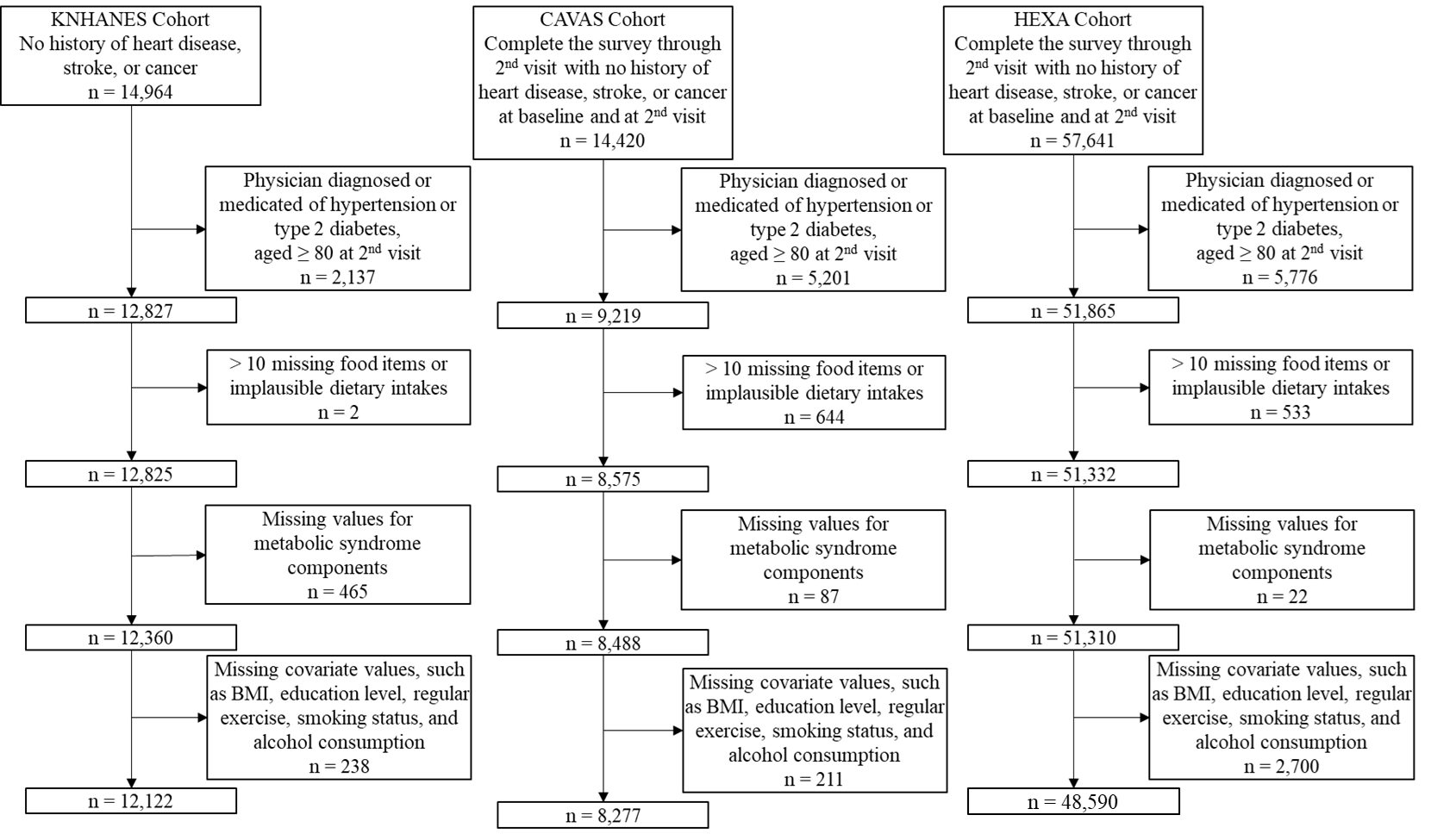


**Supplemental Table 1.** Classification of 109 food items of Korea National Health and Nutrition Examination Survey (KNHANES) and 106 food items of Cardiovascular Disease Association Study (CAVAS) and Health Examinees study (HEXA) into 39 food groups

| Food group | No. of food items | KNHANES (109 food items) | No. of food items | CAVAS, HEXA (106 food items) |
| --- | --- | --- | --- | --- |
| Total Rice | 2 | Cooked white rice; Cooked rice with multi-grains | 5 | Cooked white rice; Cooked rice with beans; Cooked rice with multi-grains; Cooked white rice only or with beans; Cooked rice with beans or with multi-grains |
| Other Rice | 3 | *Bibimbab*/Fried rice; *Gimbab*; Curry rice | - |  |
| Grain Powder | 2 | Parched cereal powder/Sikhye; Steamed corn/Roasted corn | 1 | Powdered meals/Parched cereal powder |
| Noodles | 6 | *Ramyon*/Cupped *ramyon*; Noodles/*Kalguksu*/*Udon*; *Chajangmyon*/*Champpong*; *Naengmyeon*; Dumpling; Korean pancakes (Leek pancake, *Kimchi* pancake, etc.) | 5 | *Ramyon*; *Kalguksu*/*Jangguk*-noodles/*Udon*; *Chajangmyon*/*Champpong*; *Naengmyeon*/Buckwheat noodles; Dumpling/Dumpling soup |
| Breads | 3 | Loaf bread; Bread with small red bean or cream; Castella/Cake/Chocolate pie | 3 | Loaf bread/Sandwich/Toast; Bread with small red bean; Other breads (Streusel bread/Nut cake/Castella/Cream bread) |
| Rice Cakes | 3 | *Tteokguk* (plain stick shaped-rice cake soup); Steamed rice cake/*Baekseolgi*/*Injeolmi*/*Jeolpyeon*; *Tteokbokki* | 2 | Plain stick shaped-rice cake/*Tteokguk* (plain stick shaped-rice cake soup); Other rice cakes (Steamed rice cake/*Baekseolgi*/*Injeolmi*) |
| Cornflake | 1 | Cornflake | 1 | Cornflake |
| Potatoes Starch | 4 | Steamed potatoes/Roasted potatoes; Stir-fried potatoes/Potatoes cooked in soy sauce; Steamed sweet potatoes/Roasted sweet potatoes; *Japchae* | 4 | *Muk* (Starch jelly); Potatoes (Steamed potatoes, French fries, Potato soup, Potato stew, Potato *jeon*, etc.); Sweet potatoes (Steamed sweet potatoes, *Mattang*, etc.); *Japchae* (Starch vermicelli) |
| Dessert | 3 | Snack; Cookie/Cracker; Chocolate | 3 | Cake/Chocolate pie; Cookie/Cracker/Snack; Candy/Chocolate |
| Spreads | 2 | Jam; Butter/Margarine | 1 | Jam/Honey/Margarine |
| Legumes | 6 | Beans cooked in soy sauce; Soybean paste soup; Soybean paste stew/Fast-fermented bean paste stew; *Ssamjang* (*Gochujang*, Soybean paste, Mixed paste), *Chogochujang*; Soft tofu stew, Tofu stew; Tofu/Tofu cooked in soy sauce, Stir-fried tofu | 3 | Soybeans/Soybeans cooked in soy sauce (include green beans, exclude rice with beans); Soybean paste soup/Fast-fermented bean paste/Soybean paste/*Ssamjang*; Tofu (Soft tofu, Tofu stew, and Tofu in tofu stew) |
| Soy Milk | 1 | Soy Milk | 1 | Soy Milk |
| Nuts | 2 | Peanut; Chestnut | 1 | Peanut/Almond/Pine nut |
| Green Vegetables | 7 | Spinach namul; (Ssam(rice and condiments wrapped in leaves of lettuce) vegetables/Green pepper; Vegetable salad; Boiled broccoli/Boiled cabbage; Other *namuls*; reen onion/Salad leek; Cucumber (fresh, raw) | 11 | Cabbage/Cabbage Soup; Spinach (Spinach *namul*, Soup, etc.); Lettuce (*Ssam*(rice and condiments wrapped in leaves of lettuce), Salad, etc.); Perilla leaf; Vegetable wraps/Vegetable salad (Cabbage, Lettuce, Kale, Chicory, Bok choy, Broccoli, etc.); Other green vegetables (Shepherd's purse, Beetroot, Curled mallow, Mugwort, Outer leaves, etc.); Bracken/Sweet potato stems/Taro stem; Red pepper leaves/*Chamnamul*/*Chwinamul*; Crown daisies/Leek/Water dropwort; Cucumber; Green pepper |
| Dark Yellow Vegetables | - |  | 2 | Carrot/Carrot Juice; Pumpkin/Sweet pumpkin/Pumpkin juice |
| Other Vegetables | 5 | Radish (fresh, pickled, dried); Doraji (fresh, namul); Lotus root cooked in soy sauce/Burdock cooked in soy sauce; Bean sprouts (salad, soup)/Mung bean sprouts; Pumpkin (*namul*, pancake) | 5 | Radish (Soup, Stew)/ Pickled radish; *Doraji*/*Deoduck* (kind of white root); Bean sprouts/Mung bean sprouts; Onion ; Pumpkin, immature |
| Garlic | 1 | Garlic | - |  |
| *Kimchi*, Green Leaf | 2 | *Baechukimchi*; *Kimchi* stew/Stir-fried *kimchi* | 2 | *Baechukimchi*, *Baegkimchi*, *Baechukimchi* in *Kimchi* stew; Other *Kimchi* (*Pakimchi*/*Kodulbbagi*/*Gatkimchi*) |
| *Kimchi*, White Root | 1 | Other kimchi/Geotjeori | 2 | *Kkakdugi*/Radish *Kimchi*; *Nabak* *Kimchi*/*Dongchimi* |
| Other Pickles^a^ | 1 | Pickled vegetables/Pickled cucumber | 1 | Pickled vegetables (Garlic pickles, Garlic stems, Radish pickles) |
| Mushrooms | 1 | Stir-fried mushrooms | 2 | Oyster mushroom; Other mushrooms (Wood ear mushroom, Button mushroom, *Enoki* mushroom) |
| Fruit | 12 | Strawberries; Korean melon; Watermelon; Peach; Banana; Persimmon, hard/Persimmon, dried; Tangerine; Korean pear; Apple; Orange; Grapes; Kiwi | 11 | Strawberries; Korean melon/Melon; Watermelon; Peach/Plum; Banana; Persimmon, hard/Persimmon, dried; Tangerine; Korean pear/Pear juice; Apple/Apple juice; Orange/Orange juice; Grapes/Grapes juice |
| Fruit Juice | 1 | Fruit juice | - |  |
| Tomatoes | 1 | Tomato/Cherry tomato | 1 | Tomato/Tomato juice/Cherry tomato |
| Unprocessed Red Meat | 9 | Pan roasted pork belly; Stir-fried pork/Pork *bulgogi*/Roasted pork ribs/Steamed pork ribs; Steamed pork (Boiled pork); Sweet and sour pork/ Pork cutlet; Roasted beef; Beef *bulgogi*; *Gamjatang*; *Seolleongtang*/*Gomtang*/Bone broth; Beef soup/*Yukgaejang*/*Muguk* | 7 | Pork belly; Pork, Pan roasted/Fried/Pork *bulgogi*/Meatball; Pork, steamed (Boiled pork, Pork braised in soy sauce, Pigs' feet); Steak/beef roast (Grilled ribs, Sirloin, Tenderloin, Beef *bulgogi*); Dog meat; *Tang* (*Seolleongtang*/*Gomtang*/*Galbitang*/*Doganitang*); Soup (Beef soup, *Yukgaejang*, etc.) |
| Poultry | 4 | *Samgyetang*/Chicken stew; Stir-fried chicken (Chicken ribs)/Chicken cooked in soy sauce (Chicken stews); Fried chicken; Roasted duck | 1 | Fried chicken/Whole Chicken Soup/*Samgyetang*/Chicken Stew |
| Processed Red Meat | 3 | Ham; *Budaejjigae*; *Sundae* | 2 | Processed meat (Ham, Sausage); By-products (Organ meat, *Seonji*, *Sundae*) |
| Eggs | 2 | Fried egg/Rolled egg; Boiled egg/Steamed egg | 1 | Egg/Quail egg |
| Fish | 6 | Mackerel/Pacific saury (roasted, cooked in soy sauce); Hair tail/Yellow croaker (roasted, cooked in soy sauce); Frozen Alaska pollack stew/Spicy seafood stew; Dried Alaska pollack soup; Dried anchovy/Stir-fried dried anchovies; Boiled mud-fish soup | 7 | Sliced Raw Fish; Blue-coloured back fish (Mackerel/Pacific saury/Spanish mackerel); Hair tail; Eel; Yellow croaker/Snapper/Halibut; Alaska pollack/Frozen Alaska pollack/Dried Alaska pollack; Dried anchovy/Stir-fried dried anchovies |
| Shellfish | - |  | 2 | Clam (Small ark shell/Little neck clam/Clam meat)/Whelk (including Soup, Stew, Roast, Kalguksu, Salad, etc.); Oysters (including Salted oysters) |
| Seafood | 2 | Cuttlefish/Sliced cuttlefish/Dried cuttlefish; Crab preserved in soy sauce | 3 | Cuttlefish/Dried cuttlefish/Small octopus; Crab/Crab preserved in soy sauce; Shrimp |
| Processed Seafood | 1 | Fish paste (stir-fried. soup) | 2 | Tuna, canned; Fish paste/Crab, flavoured |
| Salted Seafood | 1 | Salted shrimp/Salted squid/Salted clams | 1 | Salt-fermented fish(Salted squid, Salted intestine, Salted pollack roe, Salted shrimp, Salted anchovies, Salted clams, etc.) |
| Seaweeds | 4 | Roasted laver/Raw laver/Laver salad; Laver soup; Green laver salad/Pickled seaweed salad; Stir-fried laver stem | 2 | Laver, dried; Kelp/Sea mustard |
| Milk | 1 | Milk (low-fat, plain) | 1 | Milk |
| Dairy without Milk | 3 | Yogurt; Yoplait; Ice cream | 3 | Yogurt/Yoplait; Ice cream; Cheese |
| Carbonated Beverages | 1 | Carbonated drinks (Coke, Sprite, Fruit carbonated drinks) | 1 | Carbonated drinks (Coke, Sprite) |
| Other Beverages | - |  | 1 | Other beverages (Citron tea, Plum tea, Aloe, Persimmon punch, Ginseng tea, *Sikhye*, Jujube tea, Black herbal tea, etc.) |
| Coffee | 1 | Coffee | 1 | Coffee |
| Tea | 1 | Green tea | 1 | Green tea |
| Coffee Additives | 2 | Coffee Sugar; Coffee Cream | 2 | Coffee Sugar; Coffee Cream |
| Pizza/Hamburger | 2 | Pizza; Hamburger/Sandwich | 1 | Pizza/Hamburger |
| ***Total No of food groups*** |  | **39** |  | **39** |

^a^ ‘Other Pickles’ refers to pickled vegetables with traditional salty Korean seasonings.

**Supplemental Table 2.** Characteristics of participants in the Korea National Health and Nutrition Examination Survey (KNHANES), Cardiovascular Disease Association Study (CAVAS) and Health Examinees study (HEXA)^1^

|  | KNHANES | CAVAS | HEXA | p-diff^2^ |
| --- | --- | --- | --- | --- |
| TOTAL |  |  |  |  |
| N | 12,122 | 8,277 | 48,590 |  |
| Age (yrs) | 38.92 ± 12.05 ^a^ | 58.86 ± 8.92 ^b^ | 51.37 ± 7.95 ^c^ | < 0.001 |
| Sex (% of men) | 49.56 | 38.15 | 32.11 | < 0.001 |
| BMI (kg/m²) | 23.49 ± 3.46 ^a^ | 23.88 ± 3.02 ^b^ | 23.59 ± 2.80 ^c^ | < 0.001 |
| High school graduates (%)^3^ | 72.59 | 30.82 | 69.66 | < 0.001 |
| Regular exercise (%)^4^ | 41.75 | 23.80 | 34.43 | < 0.001 |
| Current smoker (%) | 24.12 | 13.72 | 12.91 | < 0.001 |
| Alcohol consumption (g/d) | 18.39 ± 32.66 ^a^ | 11.13 ± 30.87 ^b^ | 8.73 ± 29.19 ^c^ | < 0.001 |
| Energy intake (kcal) | 2,164.96 ± 866.43 ^a^ | 1,586.76 ± 475.82 ^b^ | 1,700.09 ± 527.45 ^c^ | < 0.001 |
| Metabolic Syndrome Prevalence (%) | 14.88 | 24.15 | 13.90 | < 0.001 |
| MEN |  |  |  |  |
| N | 4,559 | 3,158 | 15,602 |  |
| Age (yrs) | 38.42 ± 12.24 ^a^ | 60.31 ± 8.86 ^b^ | 52.07 ± 8.52 ^c^ | < 0.001 |
| BMI (kg/m²) | 24.3 ± 3.37 ^a^ | 23.7 ± 3.08 ^b^ | 24.13 ± 2.70 ^c^ | < 0.001 |
| High school graduates (%)^3^ | 71.24 | 38.35 | 78.54 | < 0.001 |
| Regular exercise (%)^4^ | 45.36 | 22.42 | 33.08 | < 0.001 |
| Current smoker (%) | 42.42 | 33.15 | 35.06 | < 0.001 |
| Alcohol consumption (g/d) | 29.70 ± 43.74 ^a^ | 25.69 ± 10.71 ^b^ | 21.95 ± 46.84 ^c^ | < 0.001 |
| Energy intake (kcal) | 2,517.56 ± 927.10 ^a^ | 1,714.9 ± 450.89 ^b^ | 1,804.92 ± 516.92 ^c^ | < 0.001 |
| Metabolic Syndrome Prevalence (%) | 19.66 | 24.76 | 19.48 | < 0.001 |
| WOMEN |  |  |  |  |
| N | 7,563 | 5,119 | 32,988 |  |
| Age (yrs) | 39.42 ± 11.92 ^a^ | 57.97 ± 8.84 ^b^ | 51.04 ± 7.64 ^c^ | < 0.001 |
| BMI (kg/m²) | 22.69 ± 3.39 ^a^ | 23.98 ± 3.08 ^b^ | 23.33 ± 2.81 ^c^ | < 0.001 |
| High school graduates (%)^3^ | 73.92 | 26.18 | 65.46 | < 0.001 |
| Regular exercise (%)^4^ | 38.20 | 24.65 | 35.07 | < 0.001 |
| Current smoker (%) | 6.15 | 1.74 | 2.44 | < 0.001 |
| Alcohol consumption (g/d) | 7.27 ± 18.69 ^a^ | 2.14 ± 10.71 ^b^ | 2.48 ± 9.78 ^c^ | < 0.001 |
| Energy intake (kcal) | 1,818.55 ± 704.91 ^a^ | 1,507.7 ± 450.89 ^b^ | 1,650.51 ± 525.13 ^c^ | < 0.001 |
| Metabolic Syndrome Prevalence (%) | 10.19 | 23.77 | 11.25 | < 0.001 |

^1^ All values are presented as mean ± SDs, or percentages.

^2^ *P* values for differences were determined using the general linear model for continuous variables and the chi-square test for categorical variables. Different lowercase letters (a, b, c) indicate statistically significant differences in continuous variables among the three groups (*P* < 0.05; Tukey’s multiple comparison test).

^3^ High school graduation or higher

^4^ ≥3 times/week and ≥30 min/session

**Supplemental Table 3.** Selected food groups of the dietary pattern (DP) and their spearman correlations with the components of MetS in the Korea National Health and Nutrition Examination Survey (KNHANES)^1^

|  | RRR dietary pattern score | DP | WC | SBP | DBP | TG | HDL-C | FBG |
| --- | --- | --- | --- | --- | --- | --- | --- | --- |
| RRR dietary pattern score | - | 0.119* | 0.674* | 0.659* | 0.667* | 0.601* | -0.487* | 0.451* |
| DP | - | - | 0.084* | 0.065* | 0.065* | 0.092* | -0.056* | 0.057* |
| Total Rice | 0.045* | 0.581* | 0.040* | 0.026* | -0.001 | 0.011 | -0.066* | 0.023* |
| Noodles | 0.058* | 0.171* | 0.046* | 0.035* | 0.052* | 0.059* | 0.012 | 0.013 |
| Rice Cakes | -0.028* | -0.264* | -0.022* | -0.020* | -0.013 | -0.019* | 0.007 | -0.021* |
| Cornflake | -0.037* | -0.243* | -0.026* | -0.029* | -0.031* | -0.034* | -0.008 | -0.017 |
| Potatoes Starch | -0.030* | -0.105* | -0.014 | -0.021* | -0.042* | -0.030* | -0.007 | -0.001 |
| Dessert | -0.043* | -0.367* | -0.027* | -0.021* | -0.012 | -0.053* | 0.028* | -0.009 |
| Spreads | -0.052* | -0.195* | -0.038* | -0.049* | -0.034* | -0.018 | 0.017 | -0.028* |
| Legumes | 0.009 | 0.238* | 0.008 | -0.003 | 0.001 | 0.008 | -0.003 | 0.018 |
| Nuts | -0.014 | 0.071* | -0.005 | 0.005 | < 0.001 | -0.021* | 0.024* | -0.007 |
| Garlic | 0.021* | 0.259* | 0.019* | 0.026* | 0.039* | 0.021* | 0.043* | 0.004 |
| Kimchi, Green Leaf | 0.058* | 0.385* | 0.050* | 0.035* | 0.027* | 0.040* | -0.022* | 0.029* |
| Kimchi, White Root | 0.036* | 0.229* | 0.045* | 0.014 | 0.016 | 0.021* | -0.006 | 0.020* |
| Mushrooms | -0.014 | -0.040* | 0.002 | -0.020* | -0.018* | -0.013 | -0.008 | -0.007 |
| Fruit | -0.028* | -0.209* | -0.006 | -0.026* | -0.033* | -0.042* | -0.016 | -0.002 |
| Fish | -0.005 | 0.112* | 0.006 | 0.001 | -0.004 | -0.016 | 0.004 | 0.001 |
| Milk | -0.010 | -0.176* | 0.022* | 0.001 | -0.011 | -0.024* | 0.024* | -0.005 |
| Carbonated Beverages | 0.071* | 0.194* | 0.050* | 0.037* | 0.037* | 0.054* | -0.024* | 0.054* |
| Tea | 0.028* | 0.127* | 0.014 | 0.023* | 0.037* | 0.013 | -0.001 | 0.013 |
| Coffee Additives | 0.007 | 0.306* | -0.002 | -0.042* | -0.010 | 0.015 | -0.056* | 0.019* |
| Pizza/Hamburger | -0.009 | -0.241* | -0.002 | 0.004 | -0.003 | -0.005 | 0.030* | < -0.001 |

RRR: reduced rank regression; WC: waist circumference; SBP systolic blood pressure; DBP: diastolic blood pressure; TG: triglyceride; HDL-C: high density lipoprotein-cholesterol; FBG: fasting blood glucose

* *P* < 0.05

^1^ Age and sex were considered for partial correlation.

**Supplemental Table 4-1.** Age- and sex-adjusted characteristics across the quintiles of the dietary pattern (DP) of the Korea National Health and Nutrition Examination Survey (KNHANES)^1^

|  | Quintile 1 | Quintile 2 | Quintile 3 | Quintile 4 | Quintile 5 | p-diff | p-trend |
| --- | --- | --- | --- | --- | --- | --- | --- |
| TOTAL |  |  |  |  |  |  |  |
| N | 2424 | 2425 | 2424 | 2425 | 2424 |  |  |
| Median (Min, Max) | 0.03 (-3.50, 0.28) | 0.44 (0.28, 0.57) | 0.71 (0.57, 0.83) | 0.98 (0.83, 1.15) | 1.42 (1.15, 4.22) |  |  |
| Age (yrs) | 32.95 ± 0.24 ^a^ | 37.19 ± 0.24 ^b^ | 39.58 ± 0.24 ^c^ | 41.92 ± 0.24 ^d^ | 44.39 ± 0.24 ^e^ | <.0001 | <.0001 |
| Sex (% of men) | 15.64 ^a^ | 31.02 ^b^ | 43.89 ^c^ | 61.41 ^d^ | 81.39 ^e^ | <.0001 | <.0001 |
| BMI (kg/m²) | 22.79 ± 0.08 ^a^ | 23.21 ± 0.07 ^b^ | 23.51 ± 0.07 ^c^ | 23.70 ± 0.07 ^c^ | 23.68 ± 0.07 ^c^ | <.0001 | <.0001 |
| Education (%) ^2^ | 75.13 ^a^ | 74.55 ^ab^ | 73.07 ^ab^ | 73.75 ^ab^ | 71.20 ^b^ | 0.0520 | 0.0058 |
| Physical activity (%) ^2^ | 45.78 ^a^ | 43.89 ^a^ | 42.44 ^a^ | 35.51 ^b^ | 32.99 ^b^ | <.0001 | <.0001 |
| Current smoker (%) | 14.09 ^ab^ | 13.50 ^a^ | 16.88 ^b^ | 20.46 ^c^ | 34.02 ^d^ | <.0001 | <.0001 |
| Alcohol consumption (g/d) | 12.05 ± 0.74 ^a^ | 11.93 ± 0.71 ^a^ | 12.46 ± 0.70 ^a^ | 16.09 ± 0.69 ^b^ | 25.96 ± 0.71 ^c^ | <.0001 | <.0001 |
| Energy intake (kcal) | 2035.35 ± 18.20 ^a^ | 1998.15 ± 17.41 ^a^ | 2015.60 ± 17.18 ^a^ | 2058.81 ± 17.10 ^a^ | 2251.39 ± 17.63 ^b^ | <.0001 | <.0001 |
| MetS Prevalence (%) | 10.63 ^a^ | 12.12 ^a^ | 14.92 ^b^ | 16.61 ^b^ | 21.17 ^c^ | <.0001 | <.0001 |
| MEN |  |  |  |  |  |  |  |
| N | 911 | 912 | 912 | 912 | 912 |  |  |
| Median (Min, Max) | 0.37 (-1.13, 0.59) | 0.75 (0.59, 0.89) | 1.01 (0.89, 1.13) | 1.27 (1.13, 1.43) | 1.68 (1.43, 4.22) |  |  |
| Age (yrs) | 33.08 ± 0.36 ^a^ | 36.62 ± 0.37 ^b^ | 39.16 ± 0.38 ^c^ | 41.53 ± 0.39 ^d^ | 42.55 ± 0.38 ^d^ | <.0001 | <.0001 |
| BMI (kg/m²) | 24.05 ± 0.11 ^a^ | 24.20 ± 0.11 ^ab^ | 24.52 ± 0.12 ^b^ | 24.25 ± 0.12 ^ab^ | 24.56 ± 0.12 ^b^ | 0.0089 | 0.0035 |
| Education (%) ^2^ | 73.07 ^ab^ | 75.17 ^ab^ | 76.98 ^a^ | 70.31 ^b^ | 71.17 ^b^ | 0.0085 | 0.1084 |
| Physical activity (%) ^2^ | 49.08 ^a^ | 48.78 ^a^ | 41.69 ^b^ | 40.67 ^b^ | 39.89 ^b^ | <.0001 | <.0001 |
| Current smoker (%) | 27.61 ^a^ | 34.07 ^b^ | 41.79 ^c^ | 49.39 ^d^ | 60.59 ^e^ | <.0001 | <.0001 |
| Alcohol consumption (g/d) | 22.18 ± 1.42 ^a^ | 23.85 ± 1.41 ^a^ | 26.85 ± 1.43 ^a^ | 33.02 ± 1.47 ^b^ | 45.12 ± 1.45 ^c^ | <.0001 | <.0001 |
| Energy intake (kcal) | 2445.02 ± 31.12 ^a^ | 2424.43 ± 30.77 ^a^ | 2425.99 ± 31.32 ^a^ | 2515.05 ± 32.08 ^a^ | 2798.62 ± 31.83 ^b^ | <.0001 | <.0001 |
| MetS Prevalence (%) | 15.40 ^a^ | 19.72 ^ab^ | 21.08 ^b^ | 23.03 ^bc^ | 27.28 ^c^ | <.0001 | <.0001 |
| WOMEN |  |  |  |  |  |  |  |
| N | 1512 | 1513 | 1513 | 1513 | 1512 |  |  |
| Median (Min, Max) | -0.06 (-3.50, 0.17) | 0.31 (0.17, 0.43) | 0.54 (0.43, 0.66) | 0.77 (0.66, 0.92) | 1.12 (0.92, 2.76) |  |  |
| Age (yrs) | 33.44 ± 0.28 ^a^ | 37.45 ± 0.29 ^b^ | 40.81 ± 0.30 ^c^ | 41.98 ± 0.30 ^d^ | 44.31 ± 0.30 ^e^ | <.0001 | <.0001 |
| BMI (kg/m²) | 22.29 ± 0.09 ^a^ | 22.55 ± 0.09 ^ab^ | 22.73 ± 0.09 ^b^ | 23.13 ± 0.09 ^c^ | 23.44 ± 0.09 ^c^ | <.0001 | <.0001 |
| Education (%) ^2^ | 73.73 ^a^ | 76.25 ^a^ | 74.36 ^a^ | 74.59 ^a^ | 68.04 ^b^ | <.0001 | 0.0008 |
| Physical activity (%) ^2^ | 42.71 ^a^ | 42.90 ^a^ | 37.37 ^b^ | 35.89 ^b^ | 30.40 ^c^ | <.0001 | <.0001 |
| Current smoker (%) | 3.86 ^a^ | 3.74 ^a^ | 5.70 ^ab^ | 6.60 ^b^ | 9.66 ^c^ | <.0001 | <.0001 |
| Alcohol consumption (g/d) | 4.14 ± 0.52 ^a^ | 5.49 ± 0.52 ^ab^ | 7.08 ± 0.53 ^b^ | 7.26 ± 0.53 ^b^ | 10.23 ± 0.54 ^c^ | <.0001 | <.0001 |
| Energy intake (kcal) | 1807.34 ± 17.98 ^a^ | 1736.39 ± 18.04 ^b^ | 1742.28 ± 18.24 ^ab^ | 1794.98 ± 18.40 ^ab^ | 1919.96 ± 18.68 ^c^ | <.0001 | <.0001 |
| MetS Prevalence (%) | 7.71 ^a^ | 8.74 ^ab^ | 11.37 ^bc^ | 12.86 ^c^ | 15.90 ^d^ | <.0001 | <.0001 |

MetS: Metabolic Syndrome

^1^ Values are adjusted for age (and sex in total), expressed as Means± SEs for the continuous variables or percentages for the categorical variables.

^2^ ≥3 times/week and ≥30 min/session

^3^ *P* values for differences were determined using the general linear model for continuous and binary categorical variables. Different lowercase letters (a, b, c, d, e) indicate statistically significant differences between groups (*P* < 0.05; Tukey’s multiple comparison test).

^4^ *P* values for linear trends were constructed by treating the median value of each group as a continuous variable.

**Supplemental Table 4-2.** Age- and sex-adjusted characteristics across the quintiles of the dietary pattern (DP) of the Cardiovascular Disease Association Study (CAVAS)^1^

|  | Quintile 1 | Quintile 2 | Quintile 3 | Quintile 4 | Quintile 5 | p-diff | p-trend |
| --- | --- | --- | --- | --- | --- | --- | --- |
| TOTAL |  |  |  |  |  |  |  |
| N | 1655 | 1656 | 1655 | 1656 | 1655 |  |  |
| Median (Min, Max) | 0.56 (-1.56, 0.75) | 0.87 (0.75, 0.97) | 1.05 (0.97, 1.13) | 1.22 (1.13, 1.34) | 1.51 (1.34, 4.36) |  |  |
| Age (yrs) | 57.67 ± 0.22 ^a^ | 59.6 ± 0.22 ^b^ | 59.96 ± 0.22 ^b^ | 59.43 ± 0.22 ^b^ | 57.66 ± 0.23 ^a^ | <.0001 | 0.7917 |
| Sex (% of men) | 15.20 ^a^ | 23.31 ^b^ | 36.72 ^c^ | 46.85 ^d^ | 68.69 ^e^ | <.0001 | <.0001 |
| BMI (kg/m²) | 23.51 ± 0.08 ^a^ | 23.73 ± 0.07 ^ab^ | 23.85 ± 0.07 ^b^ | 24.00 ± 0.07 ^b^ | 24.3 ± 0.08 ^c^ | <.0001 | <.0001 |
| Education (%) ^2^ | 44.66 ^a^ | 33.44 ^b^ | 28.51 ^c^ | 24.49 ^d^ | 23.01 ^d^ | <.0001 | <.0001 |
| Physical activity (%) ^2^ | 32.89 ^a^ | 27.3 ^b^ | 21.19 ^c^ | 19.63 ^c^ | 18.01 ^c^ | <.0001 | <.0001 |
| Current smoker (%) | 11.35 ^a^ | 10.89 ^a^ | 12.29 ^ab^ | 14.66 ^b^ | 19.45 ^c^ | <.0001 | <.0001 |
| Alcohol consumption (g/d) | 8.69 ± 0.72 ^a^ | 9.92 ± 0.71 ^a^ | 9.9 ± 0.7 ^a^ | 11.25 ± 0.71 ^a^ | 15.87 ± 0.74 ^b^ | <.0001 | <.0001 |
| Energy intake (kcal) | 1488.01 ± 11.27 ^a^ | 1508.15 ± 11.06 ^ab^ | 1539.14 ± 10.96 ^b^ | 1606.82 ± 10.99 ^c^ | 1791.69 ± 11.45 ^d^ | <.0001 | <.0001 |
| MetS Prevalence (%) | 21.11 ^a^ | 23.04 ^a^ | 23.79 ^a^ | 24.61 ^ab^ | 28.2 ^b^ | 0.0003 | <.0001 |
| MEN |  |  |  |  |  |  |  |
| N | 631 | 632 | 632 | 632 | 631 |  |  |
| Median (Min, Max) | 0.82 (-1.56, 0.97) | 1.06 (0.97, 1.14) | 1.22 (1.14, 1.3) | 1.4 (1.3, 1.51) | 1.69 (1.51, 4.36) |  |  |
| Age (yrs) | 61.19 ± 0.35 ^a^ | 61.72 ± 0.35 ^a^ | 60.56 ± 0.35 ^ab^ | 59.83 ± 0.35 ^b^ | 58.26 ± 0.35 ^c^ | <.0001 | <.0001 |
| BMI (kg/m²) | 23.72 ± 0.11 ^ab^ | 23.32 ± 0.11 ^a^ | 23.63 ± 0.11 ^ab^ | 23.87 ± 0.11 ^b^ | 23.97 ± 0.11 ^b^ | 0.0005 | 0.0047 |
| Education (%) ^2^ | 46.34 ^a^ | 41.51 ^ab^ | 38 ^bc^ | 34.61 ^bc^ | 31.28 ^c^ | <.0001 | <.0001 |
| Physical activity (%) ^2^ | 28.01 ^a^ | 23.29 ^ab^ | 20.29 ^b^ | 20.35 ^b^ | 20.18 ^b^ | 0.0024 | 0.0006 |
| Current smoker (%) | 23.44 ^a^ | 28.65 ^ab^ | 34.26 ^bc^ | 38.76 ^c^ | 40.66 ^c^ | <.0001 | <.0001 |
| Alcohol consumption (g/d) | 20.14 ± 1.76 ^a^ | 22.87 ± 1.76 ^ab^ | 25.61 ± 1.76 ^abc^ | 28 ± 1.76 ^bc^ | 31.81 ± 1.77 ^c^ | <.0001 | <.0001 |
| Energy intake (kcal) | 1627.09 ± 18.33 ^ab^ | 1578.89 ± 18.35 ^a^ | 1650.5 ± 18.3 ^b^ | 1754.21 ± 18.31 ^c^ | 1964.09 ± 18.41 ^d^ | <.0001 | <.0001 |
| MetS Prevalence (%) | 24.14 | 21.95 | 23.99 | 25.91 | 27.83 | 0.1599 | 0.0411 |
| WOMEN |  |  |  |  |  |  |  |
| N | 1023 | 1024 | 1024 | 1024 | 1024 |  |  |
| Median (Min, Max) | 0.46 (-1.19, 0.65) | 0.77 (0.65, 0.86) | 0.95 (0.86, 1.03) | 1.11 (1.03, 1.21) | 1.34 (1.21, 2.45) |  |  |
| Age (yrs) | 56.16 ± 0.27 ^a^ | 57.65 ± 0.27 ^b^ | 59.23 ± 0.27 ^bc^ | 58.89 ± 0.27 ^cd^ | 57.92 ± 0.27 ^d^ | <.0001 | <.0001 |
| BMI (kg/m²) | 23.57 ± 0.1 ^a^ | 23.78 ± 0.1 ^ab^ | 23.98 ± 0.1 ^bc^ | 24.23 ± 0.1 ^cd^ | 24.36 ± 0.1 ^d^ | <.0001 | <.0001 |
| Education (%) ^2^ | 42.49 ^a^ | 29.03 ^b^ | 23.6 ^c^ | 18.61 ^d^ | 17.18 ^d^ | <.0001 | <.0001 |
| Physical activity (%) ^2^ | 35.47 ^a^ | 27.32 ^b^ | 24.8 ^b^ | 19.4 ^c^ | 16.29 ^c^ | <.0001 | <.0001 |
| Current smoker (%) | 1.41 | 1.2 | 1.76 | 2.47 | 1.86 | 0.2274 | 0.1243 |
| Alcohol consumption (g/d) | 1.86 ± 0.34 ^a^ | 1.57 ± 0.33 ^a^ | 1.83 ± 0.33 ^a^ | 1.92 ± 0.33 ^a^ | 3.54 ± 0.33 ^b^ | 0.0002 | 0.0007 |
| Energy intake (kcal) | 1427.71 ± 13.59 ^a^ | 1413.81 ± 13.52 ^a^ | 1494.38 ± 13.55 ^b^ | 1538.71 ± 13.54 ^b^ | 1663.82 ± 13.52 ^c^ | <.0001 | <.0001 |
| MetS Prevalence (%) | 20.62 ^a^ | 22.2 ^ab^ | 23.92 ^ab^ | 26.8 ^b^ | 25.33 ^ab^ | 0.0082 | 0.0010 |

MetS: Metabolic Syndrome

^1^ Values are adjusted for age (and sex in total), expressed as Means± SEs for the continuous variables or percentages for the categorical variables.

^2^ ≥3 times/week and ≥30 min/session

^3^ *P* values for differences were determined using the general linear model for continuous and binary categorical variables. Different lowercase letters (a, b, c, d, e) indicate statistically significant differences between groups (*P* < 0.05; Tukey’s multiple comparison test).

^4^ *P* values for linear trends were constructed by treating the median value of each group as a continuous variable.

**Supplemental Table 4-3.** Age- and sex-adjusted characteristics across the quintiles of the dietary pattern (DP) of the Health Examinees study (HEXA) participants^1^

|  | Quintile 1 | Quintile 2 | Quintile 3 | Quintile 4 | Quintile 5 | p-diff | p-trend |
| --- | --- | --- | --- | --- | --- | --- | --- |
| TOTAL |  |  |  |  |  |  |  |
| N | 9,718 | 9,718 | 9,718 | 9,718 | 9,718 |  |  |
| Median (Min, Max) | 0.32 (-4.54, 0.54) | 0.7 (0.54, 0.82) | 0.92 (0.82, 1.03) | 1.14 (1.03, 1.28) | 1.5 (1.28, 6.73) |  |  |
| Age (yrs) | 51.29 ± 0.05 ^a^ | 51.43 ± 0.05 ^a^ | 51.82 ± 0.05 ^b^ | 51.73 ± 0.05 ^b^ | 50.58 ± 0.05 ^c^ | <.0001 | <.0001 |
| Sex (% of men) | 11.74 ^a^ | 19.83 ^b^ | 29.04 ^c^ | 41.56 ^d^ | 58.39 ^e^ | <.0001 | <.0001 |
| BMI (kg/m²) | 23.25 ± 0.02 ^a^ | 23.43 ± 0.02 ^b^ | 23.58 ± 0.02 ^c^ | 23.70 ± 0.02 ^d^ | 23.98 ± 0.02 ^e^ | <.0001 | <.0001 |
| Education (%) ^2^ | 77.16 ^a^ | 72.07 ^b^ | 68.40 ^c^ | 66.62 ^d^ | 64.05 ^e^ | <.0001 | <.0001 |
| Physical activity (%) ^2^ | 40.09 ^a^ | 35.67 ^b^ | 33.99 ^c^ | 31.73 ^d^ | 30.68 ^d^ | <.0001 | <.0001 |
| Current smoker (%) | 10.83 ^a^ | 11.32 ^a^ | 11.19 ^a^ | 12.63 ^b^ | 18.61 ^c^ | <.0001 | <.0001 |
| Alcohol consumption (g/d) | 7.55 ± 0.19 ^a^ | 7.97 ± 0.18 ^a^ | 8.19 ± 0.18 ^ab^ | 8.72 ± 0.18 ^b^ | 11.23 ± 0.19 ^c^ | <.0001 | <.0001 |
| Energy intake (kcal) | 1574.79 ± 3.35 ^a^ | 1579.50 ± 3.30 ^a^ | 1644.68 ± 3.28 ^b^ | 1718.79 ± 3.29 ^c^ | 1982.69 ± 3.39 ^d^ | <.0001 | <.0001 |
| MetS Prevalence (%) | 10.87 ^a^ | 12.69 ^b^ | 13.79 ^c^ | 14.88 ^d^ | 17.24 ^e^ | <.0001 | <.0001 |
| MEN |  |  |  |  |  |  |  |
| N | 3,120 | 3,121 | 3,120 | 3,121 | 3,120 |  |  |
| Median (Min, Max) | 0.63 (-2.85, 0.83) | 0.95 (0.83, 1.05) | 1.14 (1.05, 1.24) | 1.36 (1.24, 1.5) | 1.72 (1.5, 6.46) |  |  |
| Age (yrs) | 52.70 ± 0.10 ^a^ | 52.68 ± 0.10 ^a^ | 52.69 ± 0.10 ^a^ | 51.77 ± 0.10 ^b^ | 50.50 ± 0.10 ^c^ | <.0001 | <.0001 |
| BMI (kg/m²) | 24.00 ± 0.03 ^a^ | 24.03 ± 0.03 ^a^ | 24.08 ± 0.03 ^a^ | 24.24 ± 0.03 ^b^ | 24.31 ± 0.03 ^b^ | <.0001 | <.0001 |
| Education (%) ^2^ | 83.19 ^a^ | 79.85 ^b^ | 78.16 ^bc^ | 76.44 ^cd^ | 75.06 ^d^ | <.0001 | <.0001 |
| Physical activity (%) ^2^ | 37.07 ^a^ | 34.39 ^b^ | 31.98 ^c^ | 30.79 ^c^ | 31.16 ^c^ | <.0001 | <.0001 |
| Current smoker (%) | 27.23 ^a^ | 30.01 ^b^ | 34.08 ^c^ | 39.01 ^d^ | 44.98 ^e^ | <.0001 | <.0001 |
| Alcohol consumption (g/d) | 17.92 ± 0.54 ^a^ | 20.52 ± 0.54 ^b^ | 21.70 ± 0.54 ^bc^ | 23.24 ± 0.54 ^c^ | 26.38 ± 0.54 ^d^ | <.0001 | <.0001 |
| Energy intake (kcal) | 1631.95 ± 5.54 ^a^ | 1660.81 ± 5.54 ^b^ | 1728.09 ± 5.54 ^c^ | 1856.69 ± 5.53 ^d^ | 2147.05 ± 5.55 ^e^ | <.0001 | <.0001 |
| MetS Prevalence (%) | 17.05 ^a^ | 17.88 ^ab^ | 19.33 ^bc^ | 20.78 ^cd^ | 22.38 ^d^ | <.0001 | <.0001 |
| WOMEN |  |  |  |  |  |  |  |
| N | 6,598 | 6,597 | 6,598 | 6,597 | 6,598 |  |  |
| Median (Min, Max) | 0.24 (-4.54, 0.45) | 0.59 (0.45, 0.72) | 0.82 (0.72, 0.92) | 1.02 (0.92, 1.15) | 1.34 (1.15, 6.73) |  |  |
| Age (yrs) | 50.71 ± 0.06 ^a^ | 50.89 ± 0.06 ^a^ | 51.28 ± 0.06 ^b^ | 51.42 ± 0.06 ^b^ | 50.87 ± 0.06 ^a^ | <.0001 | <.0001 |
| BMI (kg/m²) | 23.01 ± 0.02 ^a^ | 23.15 ± 0.02 ^b^ | 23.28 ± 0.02 ^c^ | 23.45 ± 0.02 ^d^ | 23.77 ± 0.02 ^e^ | <.0001 | <.0001 |
| Education (%) ^2^ | 72.72 ^a^ | 68.60 ^b^ | 64.66 ^c^ | 61.56 ^d^ | 59.75 ^e^ | <.0001 | <.0001 |
| Physical activity (%) ^2^ | 41.11 ^a^ | 36.36 ^b^ | 34.49 ^c^ | 32.35 ^d^ | 31.06 ^d^ | <.0001 | <.0001 |
| Current smoker (%) | 1.94 ^a^ | 2.54 ^b^ | 2.23 ^ab^ | 2.26 ^ab^ | 3.21 ^c^ | <.0001 | <.0001 |
| Alcohol consumption (g/d) | 2.17 ± 0.08 ^a^ | 2.39 ± 0.08 ^a^ | 2.28 ± 0.08 ^a^ | 2.42 ± 0.08 ^a^ | 3.15 ± 0.08 ^b^ | <.0001 | <.0001 |
| Energy intake (kcal) | 1573.41 ± 4.04 ^a^ | 1518.55 ± 4.04 ^b^ | 1615.90 ± 4.04 ^c^ | 1666.03 ± 4.04 ^d^ | 1878.64 ± 4.04 ^e^ | <.0001 | <.0001 |
| MetS Prevalence (%) | 8.54 ^a^ | 10.11 ^b^ | 11.12 ^c^ | 12.46 ^d^ | 14.02 ^e^ | <.0001 | <.0001 |

MetS: Metabolic Syndrome

^1^ Values are adjusted for age (and sex in total), expressed as Means± SEs for the continuous variables or percentages for the categorical variables.

^2^ ≥3 times/week and ≥30 min/session

^3^ *P* values for differences were determined using the general linear model for continuous and binary categorical variables. Different lowercase letters (a, b, c, d, e) indicate statistically significant differences between groups (*P* < 0.05; Tukey’s multiple comparison test).

^4^ *P* values for linear trends were constructed by treating the median value of each group as a continuous variable.
